# Book Review: Stereotypes and Language Learning Motivation: A Study of L2 Learners of Asian Languages

**DOI:** 10.3389/fpsyg.2021.736140

**Published:** 2021-07-30

**Authors:** Dongfang Hu

**Affiliations:** College of Media and International Culture, Zhejiang University, Hangzhou, China

**Keywords:** stereotypes, language learning, motivation, L2, Asian languages

It is conceptualized that “stereotypes serve as an indispensable cognitive device to aid the processing of an incessant flow of information that we receive in our daily life” (p. 1). In the context of foreign language education, this issue becomes more ubiquitous and germane in that language learners need to not only master another language but also enhance their understanding of the idiosyncratic nature of the target language country, its cultures, and community. Consequently, language educators need to serve an essential role in fostering their students' nuanced and comprehensive appreciation of the world surrounding them. Nonetheless, the associative relationships between language learners' country stereotypes and their language learning motivation have remained scantly. To bridge this gap, Larisa Nikitina's monograph, titled *Stereotypes and Language Learning Motivation: A Study of L2 Learners of Asian Languages*, aims to pursue this line of research by scrutinizing the correlations between country stereotypes and L2 motivation, drawing on a robust and systematic methodology. What makes this book picturesque is its focus on mental images that learners of non-European languages hold about Asian languages with respect to their L2 motivation.

This monograph consists of an Introduction and five chapters. Chapter 1, entitled *Stereotypes as a Research Focus*, overviews the origins of stereotypes and appraises the pertinent studies on this construct with a focus on country and national stereotypes as an interdisciplinary construct across diverse fields of study. Regarding the methodological issues, this chapter conceptualizes this construct and its related approaches and elaborates on stereotype content, stereotype formation, stereotype processes, stereotype accuracy, as well as individual and consensual stereotypes. Furthermore, this chapter overviews the methodological approaches by explaining the structured and unstructured approaches, measuring instruments, and assessing stereotype valence and salience. The chapter closes with its detailed account of the research focus, objectives, and methodologies on stereotypes held by language learners.

Chapter 2 reviews the extant literature on attitudes, motivation, and stereotypes in L2 research, aiming to systematically, theoretically, and empirically connect the dots. Drawing on Vygotsky (1934) conceptualization, Nikitina argues that “word meaning and word sense form a dyadic unity” (p. 35), so she considers this theoretical foundation to underpin her study. What seems to be missing in this chapter is to elaborate on how complex dynamic systems theory (CDST) sheds light on the L2 motivation, as an intricate and dynamic phenomenon (Dörnyei et al., 2015).

Chapter 3 justifies the need to utilize a mixed-methods research design to explore the links between motivation, attitudes, and country stereotypes. Set in the Malaysian context, this study employed a convenience sampling method that included 130 undergraduate students learning Mandarin, Japanese, Korean, Thai, Myanmar, and Vietnamese. The chapter clearly elucidates the content analysis and coding processes as well as the assessments of stereotype salience and accuracy. However, I expected to see how the author draws on the principles of qualitative analysis by referring to such important issues as confirmability, credibility, transferability, member checking, and audit trail to solidify the findings and pave the way for a better replication and extrapolation (Lincoln and Guba, 1985). Furthermore, had the author added some teacher-student interpersonal factors (rapport, credibility, confirmation, immediacy, etc.) or psychological variables, the findings could have been more insightful. In the remainder of this chapter, the author describes the quantitative data analysis by running exploratory factor analysis, Spearman correlation test, and regression analysis to scrutinize the correlations between motivation and language learners' mental images of the target language.

The penultimate chapter unravels the findings. It reveals the consensual stereotypes of the six Asian target language countries and elucidates their favourability and salience parameters. Not only does Chapter 4 report on the content of larger categories of images of these countries, but it also sheds light on the interconnectedness of these constructs being informed by both qualitative and quantitative analyses. I recommend that, in the new edition of this monograph, the author add a colorful image to show the interconnections of these variables by reporting the outputs through AMOS, MPlus, and LISREL. The present evidence-based study focused on the relationships between the language students' mental images of the target language countries and their L2 motivation, so it is expected that the predictive role of stereotypes can be measured. The closing chapter summarizes the key findings, provides a range of methodological, theoretical, and pedagogical implications, and suggests some new strands for future research.

This book has some merits. First of all, it taps upon an underappreciated area of inquiry in the Asain context which *per se* provides a treasure trove of information. Secondly, the monograph is methodologically rigorous and pedagogically illuminating, paving the way for interested researchers to continue this line of inquiry. Thirdly, the review of the theoretical underpinnings and the appraisal of the previous research can offer the readers a refreshing outlook. Fourthly, discussing the findings gleaned from the participants from six countries deeply broadens our perspectives toward the complex and dynamic nature of L2 motivation and stereotypes. All in all, this monograph is an opportune contribution for researchers, teachers, and learners who are interested in the dynamic interplay of “pictures in the heads” and L2 motivation.

## Author Contributions

DH was the sole author of this work.

## Conflict of Interest

The author declares that the research was conducted in the absence of any commercial or financial relationships that could be construed as a potential conflict of interest.

## Publisher's Note

All claims expressed in this article are solely those of the authors and do not necessarily represent those of their affiliated organizations, or those of the publisher, the editors and the reviewers. Any product that may be evaluated in this article, or claim that may be made by its manufacturer, is not guaranteed or endorsed by the publisher.
